# TCR-NP: a novel approach to prioritize T-cell Receptor repertoire network properties

**Published:** 2024-12-30

**Authors:** Shilpika Banerjee, Phi Le, Hai Yang, Li Zhang, Tao He

**Affiliations:** 1 Department of Mathematics, San Francisco State University, San Francisco, CA 94132, USA; 2 Department of Medicine, University of California San Francisco, San Francisco, CA 94143 USA; 3 Helen Diller Family Comprehensive Cancer Center, University of California, San Francisco, San Francisco, CA 94143, USA; 4 Department of Epidemiology & Biostatistics, University of California San Francisco, San Francisco, CA 94143, USA

## Abstract

T-cell Receptors (TCRs) play a pivotal role in antigen recognition and binding, and their sequence similarity significantly impacts the breadth of antigen recognition. Network analysis is employed to explore TCR sequence similarity and investigate the architecture of the TCR repertoire. Network properties hence could be utilized to quantify the structure of the TCR network. However, the heterogeneous nature of TCR network properties poses challenges in performing statistical learning across subjects directly, particularly when assessing their relationship with disease states, clinical outcomes, or patient characteristics. To overcome this challenge, a powerful method is developed, TCR-NP (TCR Network properties Prioritization), that aggregates the raw heterogeneous network properties and conducts grouped feature selection using a pseudo-variables-assisted penalized group Lasso model. Unlike the traditional parameter-tuning using cross-validation, a novel tuning strategy is introduced by incorporating permutation and pseudo-variables to improve the selection performance. The effectiveness of the proposed method is demonstrated through comprehensive evaluation, including simulation studies and real data analysis. By comparing the performance of the different approaches, the advantages of the proposed methodology in capturing the underlying relationships between TCR network properties and clinical outcomes or patient characteristics are highlighted.

## Introduction

T-cells are one of the key components of the adaptive immune system^[1]^. T-cell Receptors (TCRs)^[1,2]^ are a group of protein complexes on the surface of T-cells. TCRs recognize and bind to specific antigen peptides^[3]^ found on abnormal cells or potentially harmful pathogens. Once the TCRs bind to the pathogens, the T-cells attack these cells and help the body fight infection, cancer, or other diseases. TCR repertoires, which are continually shaped throughout the lifetime of an individual in response to pathogenic exposure, can serve as a fingerprint of an individual's current immunological profile. The protein structures of TCRs determine the binding between TCRs and antigen peptides^[4]^. Thus, the similarity among TCR sequences directly influences the antigen peptide recognition breadth. Network analysis, where TCR clones are represented by vertices and connected if similar in sequences (distance is less than a particular number) by using some sequence similarity measures (e.g., Hamming distance, Levenshtein distance^[5]^, etc.), was used as a novel perspective to study TCR clusters and their binding to antigen peptides. As the binding patterns will eventually impact the high-level responses, the aim is to use network structure as a special layer of information to investigate its potential connection to clinical outcome or disease status, as evidenced in existing literature^[6]^. For example, it was observed that lung cancer patients with focused TCR repertoires and complex network connections attained significantly longer overall survival (OS) than those with smaller clusters^[7]^. Therefore, quantitative analysis of the TCR repertoire network properties has the potential to provide a better understanding of the immune landscape involving T cell responses. However, network properties are highly heterogeneous, as they can be measured at node and cluster levels, and networks differ in the number of nodes and clusters. Thus, it is challenging to perform statistical inference or machine learning directly on the TCR network properties to study their relationship with clinical outcomes.

A flexible and efficient approach was proposed to prioritize TCR Network Properties (TCR-NP) by leveraging extracted features from the heterogeneous network properties to assess their relationship with the outcome of interest, while incorporating the group structure based on the nature of the features. As an initial investigation, we propose extracting simple summary statistics (e.g., min, Q1, mean, median, Q3, max) from the network property values since they can be easily calculated and carry the key signatures of a distribution. Since the extracted features are naturally grouped by network property, TCR-NP is constructed on the Group Lasso model^[8]^, a classical statistical method that offers several advantages in feature selection and prioritization. Specifically, it promotes group-level selection and addresses multicollinearity issues by selecting or excluding the entire groups (network properties), which is particularly beneficial when dealing with highly correlated variables (often found within the same network property). Moreover, it can handle the high-dimensional data (small sample, a large number of features commonly seen in TCR network data) efficiently (via L1 regularization^[9]^), resulting in a sparse solution that will facilitate the subsequent interpretations. Additionally, it can be applied to different response types (e.g., categorial, quantitative, and time-to-event), which will fulfill the needs of different application scenarios.

Instead of using the commonly used cross-validation (CV)^[10]^ technique, it was proposed to utilize pseudo-variables to assist the selection in the Group Lasso model, inspired by Yang et al.^[11]^. Traditional CV tuning typically minimizes prediction errors, which are indirect measures of selection performance. In contrast, pseudo-variables, generated through permutation as artificial unrelated features, serve as 'known negatives'. The goal is to select features with stronger association signals than the pseudo-variables, which is a more direct approach to improve selection performance. Such a strategy enhances precision by minimizing false positives, resulting in a condensed set of strongly associated features. Moreover, acting as a filter, pseudo-variables contribute to a more robust model, capable of handling variations and maintaining performance in noisy datasets.

In this paper, extensive simulation studies under different scenarios were conducted to demonstrate the efficacy of TCR-NP. Performance measures, including F-1 score, False Discovery Rate (FDR), sensitivity, and stability were calculated for each of the four following methods: permutation-assisted Group Lasso (P-Group Lasso), cross-validation tunned Lasso (CV-Lasso), cross-validation tunned Group Lasso (CV-Group Lasso), and permutation-assisted Lasso (P-Lasso). The proposed methods were also applied to a lung cancer TCR data for illustration.

## Materials and methods

### Network analysis and network properties

**Fig. 1:**
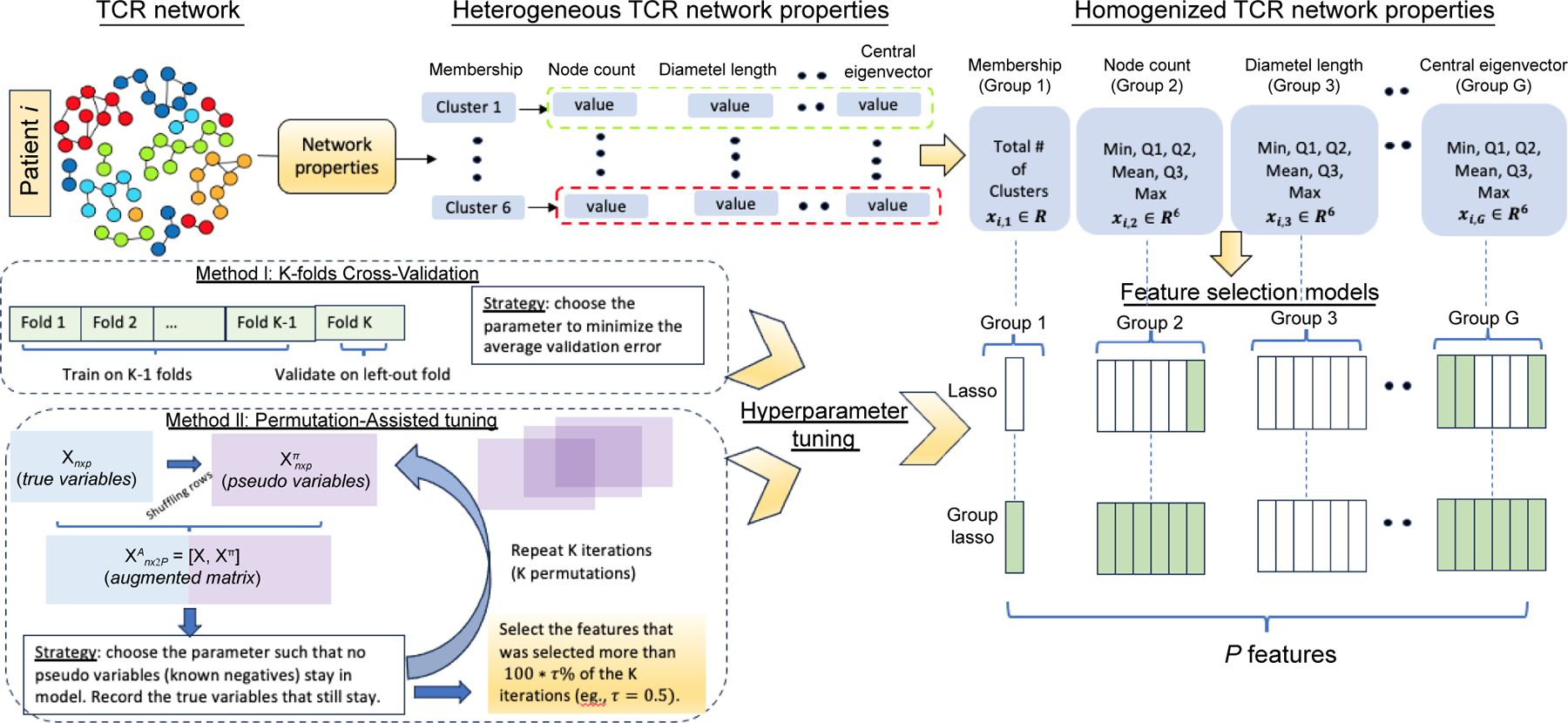
Proposed pipeline. Top: Derivation of the network properties to network property features; Bottom left: Cross-Validation (CV) tuning and permutation-assisted tuning; Bottom right: Summary of the feature selection models (Lasso and Group Lasso).

A matrix of pairwise distance of amino acid sequences was calculated for each sample based on Levenshtein distance^[5]^. Then, a TCR network can be generated by connecting the amino acid sequences (nodes) with a distance less than or equal to 1 (allowing a maximum of 1 amino acid difference among sequences). A cluster of a network represents a group of clones that are similar in sequence, and here, clusters are only considered with at least two clones (nodes). Based on the network generated, several quantitative properties, such as the number of clusters, diameter, assortativity, etc, are calculated (Table 1). Network analysis was performed using the R package NAIR^[12]^. As mentioned earlier, within each TCR repertoire for each sample, there are different numbers of clusters, each corresponding to its properties. Therefore, for each property, the property dimension varies amongst samples. To tackle those issues, descriptive summary statistics for all the TCR repertoire network properties were derived and considered them as *network property features* (Fig. 1, top) for each patient. These summary statistics contain minimum, 1^st^ quartile $\left(Q_{1}\right)$, median, mean, 3^rd^ quartile $\left(Q_{3}\right)$, and maximum values. This approach helps obtain the TCR *network property features* for each patient (Table 2). Those results are independent of the number of collected TCRs for each patient and the number of clusters for each network, making the input structure the same across patients and suitable for making statistical inferences at the patients' level.

### Group Lasso model

Assume a total of G network properties are considered, where the g-th property generated $v_{g}$ *network property features* by using summary statistics of the network property, g=1,…,G. Define $x_{i}={\left(x_{i,1}^{T},\cdots ,x_{i,G}^{T}\right)}^{T}$ as the network property features generated from i-th patienťs TCR repertoires, where the $x_{i,g}\in R^{vg}$ represents the features from g-th property, with $\sum_{g=1}^{G}v_{g}=P$, i=1,…,n. Let $y=\left(y_{1},\ldots ,y_{n}\right)$ be a binary response of interest, i.e., $y_{i}\in \left\{0,1\right\}$, i=1,…,n. The binary response could be disease status, response to treatment, prolonged survival, etc. We assume the relationship between the response variable and network property features follows a logistic regression model:

$$\mathrm{Pr}\left(y_{i}=1\left|x_{i}\right.\right)=\frac{\exp\left(\eta_{\beta }\left(x_{i}\right)\right)}{1+\exp\left(\eta_{\beta }\left(x_{i}\right)\right)}$$

where,

$$\eta_{\beta }\left(x_{i}\right)=\beta_{0}+\beta_{1}^{T}x_{i,1}+\cdots +\beta_{G}^{T}x_{i,G}$$

The above equation represents a linear combination of network property features. $\beta ={\left(\beta_{0},\beta_{1}^{T},\cdots ,\beta_{G}^{T}\right)}^{T}$ is the logistic regression coefficient where $\beta_{g}\in R^{v_{g}}$ is the coefficient vector for $v_{g}$ network features generated by g-th property, g=1,…,G. The goal is to identify the network properties that are associated with the response variable, i.e., identify the property feature groups with $\beta_{g}\neq 0$. The Group Lasso method (Fig. 1, bottom right) is well-fitted to the problem due to the group structure among variables and the need for shrinkage. It can efficiently shrink the coefficients of less important groups to exactly zero for high-dimensional data, while the group with nonzero coefficients could stand out and are considered the most important properties associated with the response variable. The solution of the logistic group Lasso model corresponds to an optimization problem by minimizing the objective function:

$$L_{\lambda }\left(\beta \right)=-\sum_{i=1}^{n}\left[y_{i}\eta_{\beta }\left(x_{i}\right)-\log\left(1+\exp\left(\eta_{\beta }\left(x_{i}\right)\right)\right)\right]+\lambda \sum_{g=1}^{G}s\left(v_{g}\right){\left\|\beta_{g}\right\|}_{2}$$

where, $s\left(v_{g}\right)$ is the penalty for gth set and by default is set to $\sqrt{v_{g}}$ for group Lasso model (i.e., the larger penalty for the larger set), ${\left\|\beta_{g}\right\|}_{2}$ represents the ${\text{L}}_{2}$ norm of the vector $\beta_{g}$, and λ≥0 is a tuning parameter controlling the amount of shrinkage. A large λ promotes heavier shrinkage, i.e., more coefficient vectors $\beta_{g}$ shrink to zero. In the extreme case, when λ=0, the solution of the optimization problem is the same as the logistic regression coefficient, while λ=∞ gives $\left(\beta_{1}^{T},\cdots ,\beta_{G}^{T}\right)=0$, i.e., shrinking all coefficients vectors to zero. For a given λ an estimate of $\hat{\beta }\left(\lambda \right)$ can be obtained by solving the optimization.

### A novel approach for group feature selection

Selecting the right tuning parameter λ is crucial for improving the performance and robustness of a model. Shrinkage techniques like Lasso and Group Lasso typically use K-fold cross-validation to identify the optimal value of the tuning parameter λ from a range of different λ values. In this method, the dataset is divided into K equal folds. For each candidate λ value, the model is trained using K−1 folds and validated on the remaining fold. This process is repeated for each λ value and the optimal λ is chosen based on minimizing the average loss, such as mean square error for Gaussian response or deviance for a binary response, across all validation folds. However, the average loss that guides the selection, is not a direct measure of the selection performance. In the past decade, pseudo-variables have been used to improve the performance of variable selection^[13,14]^. Inspired by Yang et al.^[11]^, where pseudo-variables were utilized to assist the variable selection in the Lasso model and applied to genome-wide association studies, the pseudo-variable assisted tuning procedure was developed on the proposed Group Lasso model (Fig. 1, bottom left) to identify the important network properties associated with the clinical outcome.

First, the G groups of pseudo-features are introduced $x_{i}^{\pi }={\left(x_{i,\left(G+1\right)}^{T},\cdots ,x_{i,2G}^{T}\right)}^{T}$, which is generated by a permutation π (i.e. randomly shuffling the rows of the original matrix). Hence the augmented features $x_{i}^{A}$ include both the original grouped variables $x_{i}$ and the pseudo-grouped variables $x_{i}^{\pi }$.


$$x_{i}^{A}={\left(x_{i,1}^{T},\cdots ,x_{i,G}^{T},x_{i,\left(G+1\right)}^{T},\cdots ,x_{i,\left(2G\right)}^{T}\right)}^{T}.$$

The updated logistic regression model becomes:

$$\mathrm{Pr}\left(y_{i}=1\left|x_{i}^{A}\right.\right)=\frac{\exp\left(\eta_{\beta^{A}}\left(x_{i}^{A}\right)\right)}{1+\exp\left(\eta_{\beta^{A}}\left(x_{i}^{A}\right)\right)}$$

where,

$$\eta_{\beta^{A}}\left(x_{i}^{A}\right)=\beta_{0}+\beta_{1}^{T}x_{i,1}+\cdots +\beta_{2G}^{T}x_{i,2G}$$

represents a linear combination of augmented features. The logistic Group Lasso estimator ${\hat{\beta }}_{\lambda }^{A}$ for this augmented design matrix is derived by minimizing the below objective function.


$$L_{\lambda }\left(\beta^{A}\right)=-\sum_{i=1}^{n}\left[y_{i}\eta_{\beta^{A}}\left(x_{i}^{A}\right)-\log\left(1+\exp\left(\eta_{\beta^{A}}\left(x_{i}^{A}\right)\right)\right)\right]+\lambda \sum_{g=1}^{2G}s\left(v_{g}\right){\left\|\beta_{g}\right\|}_{2}$$

Since the pseudo-variables are generated by permutations, their group sizes are the same as the original ones, i.e. $v_{G+g}=v_{g}$, g=1,…,G. In the above equation, the tuning parameter λ≥0 controls the amount of penalization (i.e., how many groups have non-zero coefficient vectors). More explicitly, 2*G* groups have non-zero coefficient vectors when λ=0. As λ increase, more groups are excluded (i.e., coefficient vectors are shrunk to zeros) from the model until every group is excluded when λ is large enough, following the fashion that more important ones stay in the model longer when λ increases. Therefore, the magnitude of λ reflects the importance of the variable: if a group still has a nonzero coefficient when λ is relatively large, this group is considered more important, compared to the ones that have been shrunk to zeros. Along with this idea, we define an importance metric for the g-th variable group.


$$W_{g}=sup\left\{\lambda :{\hat{\beta }}_{g}^{A}\left(\lambda \right)\neq 0\right\};g=1,\cdots ,2G$$

The group-variable selection procedure given below assumes that true active grouped variables are more likely to stay in the model than the pseudo-grouped variables (known noises) when the penalty *λ* increases. Define $C_{\pi }=max_{\left(G+1\right)⩽g⩽2G}\left(W_{g}\right)$ for the permutation copy π, i.e., the largest importance score among the pseudo groups. This can serve as a benchmark to separate the true active group variables from the pseudo group variables. We want to select the true groups that are more important than the strongest signal among the pseudo groups. Specifically, the selection of groups under a particular permutation π is defined as:

$${\hat{S}}_{\pi }=\left\{g:W_{g}>C_{\pi },g=1,\cdots ,G\right\}.$$

The selection process involves iteratively creating K different permutation copies (e.g. K=50) to evaluate the frequency of selection for each of the G groups across these K permutations. A group will be selected if its selection frequencies out of the K permutations are greater than a threshold τ.

### Lung cancer data

The TCR repertoire sequencing data of 65 patients enrolled in the Phase I trial NCT01693562, 14 September, 2012) of durvalumab was included for this analysis. Patients with OS ≥ 20.3 months are categorized into the longer overall survival group and patients with OS < 20.3 months are categorized into the shorter overall survival group, where the median overall survival was 20.3 months. The bulk TCR beta chain sequencing was done for each blood sample (two samples per patient including baseline and post-treatment) by the Invitrogen Qubit dsDNA HS assay (Thermo Fisher Scientific). The median number of unique clonotypes was 4,994 (ranging from 403 to 17,876). The clinical characteristics of the patients and sequencing information are as reported in the study by Naidus et al.^[7]^.

### Simulation strategy

To demonstrate the performance of the proposed method on TCR data, an efficient simulation approach is proposed to generate TCR network properties based on real data (Supplementary Figure S1). Firstly, the values of the network properties were computed based on the observed data. The correlation structure of the properties was also estimated using the observed data. Secondly, the empirical distributions for cluster size were approximated (using log-normal distribution with estimated parameters). Thirdly, based on the estimated distributions and correlation structure the artificial data was simulated to mimic the real data. This process was repeated to generate network properties for a sample of n patients. Finally, the summary statistics were extracted for each of the 11 properties and aggregated these summary statistics to generate 70 network features as listed in Table 2. Besides the 70 network features, additional variables were simulated using Uniform(0,1) distribution to mimic appliable variables from other sources, resulting in a total of P features.

To simulate the response variable, it is assumed there are four non-observed causal variables $\left\{Z_{1},Z_{2},Z_{3},Z_{4}\right\}$, corresponding to four different network properties, where each causal variable is a linear combination of percentiles from the distribution of the corresponding property (Supplementary Fig. S2). The four causal properties generate 25 observed network property features (shown in bold font in Table 2), which are considered as (indirect) causal variables in the simulation studies. The rest of the *P-25* network features are then considered as non-casual variables. The aim is to evaluate how well the proposed method could determine those 25 causal variables. The response variable is generated using the logistic regression model:

$$\mathrm{Pr}\left(y_{i}=1\right)=\frac{\exp\left\{\eta_{\alpha }\left(Z_{i}\right)\right\}}{1+\exp\left\{\eta_{\alpha }\left(Z_{i}\right)\right\}}$$

where, $\eta_{\alpha }\left(Z_{i}\right)$ is a function of the four causal variables, which can be either a linear or nonlinear function. Then $y_{i}$ is generated *via* a random sample from Bernoulli distribution with $\mathrm{Pr}\left(y_{i}=1\right)$ for i=1,…,n. The simulation is repeated N=100 times under each of the 12 different scenarios (Table 3) with various sample size n, various dimension parameter P, balanced or unbalanced response, and a linear or nonlinear relationship in $\eta_{\alpha }\left(Z_{i}\right)$.

### Performance evaluation criteria

Performance measures, including sensitivity, False Discovery Rate (FDR), F-1 score, and stability, are used to evaluate the various feature selection models. Sensitivity is defined as the proportion of correctly identifying causal variables among the total 25 causal variables in a single iteration and higher sensitivity is preferred. FDR is defined as the frequency of false-positive findings among all variables selected and a lower value is preferred. F-1 score is the harmonic mean of the sensitivity and precision (1-FDR), i.e. $2\times \left(\text{Precision}\times \text{Sensitivity}\right)/\left(\text{Precision}+\text{Sensitivity}\right)$. It is a balanced measure between sensitivity and precision of the model and a higher value is preferred. The average sensitivity, FDR, and F-1 among the N=100 simulation replicates were calculated and reported. To estimate the stability of a variable selection model, all pairwise combinations of the N=100 selected variable lists from all iterations are considered. For each pair, the stability of the two lists of selected variables is determined using the Jaccard's index given as $J\left(A_{i},A_{j}\right)=\frac{\left|A_{i}\cap A_{j}\right|}{\left|A_{i}\cup A_{j}\right|}$, where Ai, $Aj(i\neq j;i,j\in \left\{1,2,\ldots ,N\right\}$ are the list of variables selected in the *i*-th and *j*th iteration respectively, $\left|\cdot \right|$ denotes the cardinality of the set. Jaccard's index takes values between 0 and 1, where a zero value indicates the two lists do not overlap, and a one Jaccard index means the two lists contain exactly the same variables (i.e., very stable). The average of all pairs is used as the stability value for that method.

## Results

### Real data analysis results

**Fig. 2:**
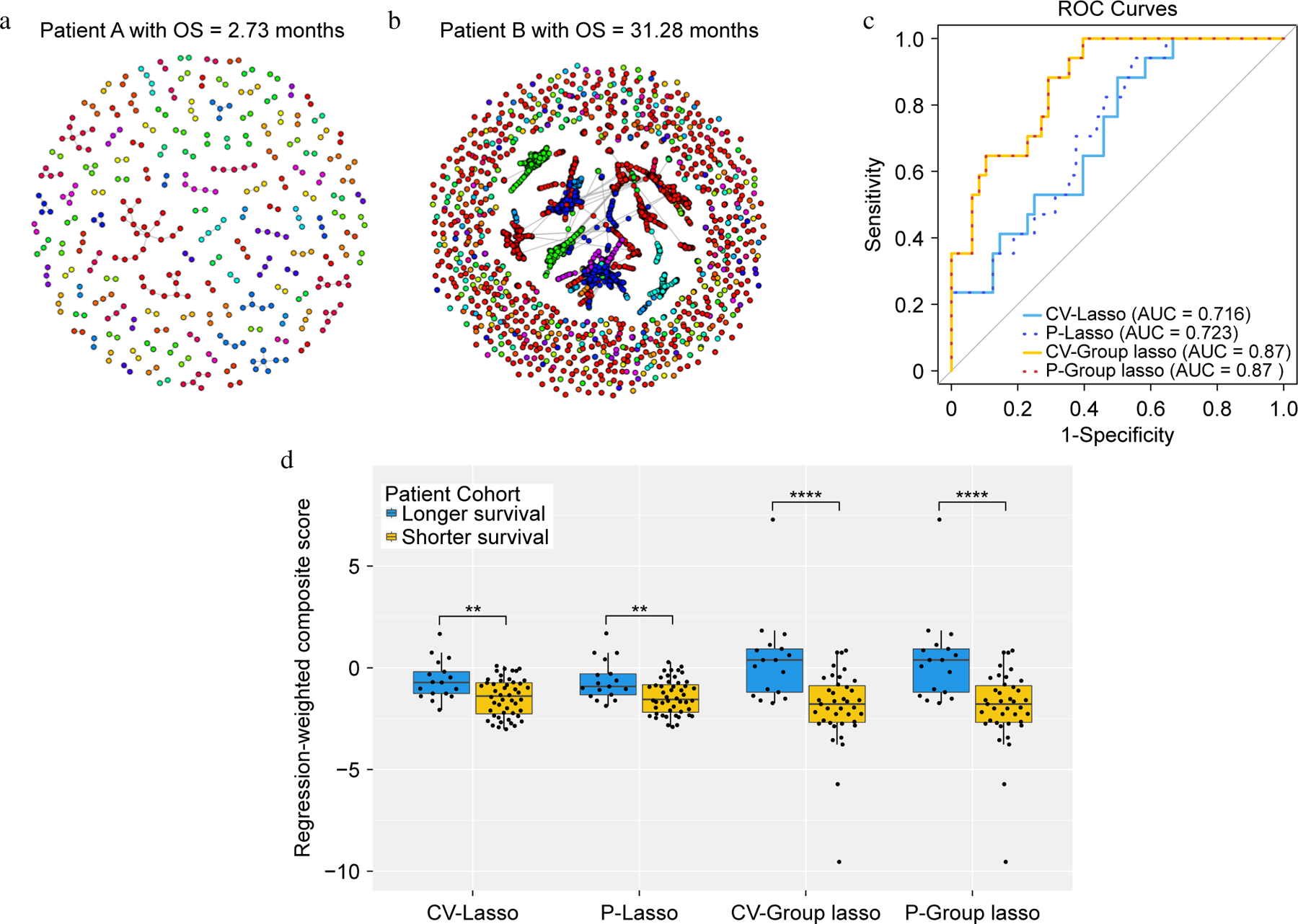
The results for the lung cancer dataset. (a) and (b) Networks for two representative patients. Within each network figure, each node represents TCR and nodes are connected if their distance is less than or equal to 1. (c) ROC curves for each of the approaches. (d) Boxplots of the composite scores.

TCR repertoire network analysis was conducted for each of 65 lung cancer patients^[7]^. Figure 2a and b illustrate the network for two representative patients. The number of TCR clusters in each patient ranged from 15 to 883 per patient with a median of 271. Eleven network properties for each TCR cluster were evaluated (Table 1) and 70 *network property features* derived (Supplementary Table S2) by obtaining summary statistics of each network property for each patient (Fig. 1 & Table 2). The summary statistics consist of descriptive information like minimum, 1^st^ quartile, median, mean, 3^rd^ quartile, and maximum values and the proportion of *NA* if it exists. The existence of NA values is due to not being able to evaluate for a particular cluster structure. For example, assortativity, transitivity, central Eigen, and central closeness are all NA when there are two nodes in one cluster (Supplementary Fig. S1). All extracted features are then standardized, following common practice. CV-Lasso, P-Lasso, CV-Group Lasso, and P-Group Lasso models were then applied with the corresponding parameters listed in Supplementary Table S1. The significant *network property features* identified by the P-Lasso model were a subset of those from the CV-Lasso model, aligning with the known tendency of permutation-assisted tuning to reduce false positives (Table 4). The consistency between P-Group Lasso and CV-Group Lasso results, including identical prediction outcomes (AUCs), strengthen findings (Fig. 2c). Both models selected all features from the most significant network properties, resulting in a higher AUC (0.87) than CV-Lasso and P-Lasso (Fig. 2c). Furthermore, composite scores were calculated using the linear combination of the model coefficients times the corresponding selected features in the logistic regression model. The weighted composite scores were compared between longer and shorter survival (overall survival greater than or less than the median overall survival, respectively) by two-sample t-test. It was found that the differences in scores between longer and shorter survival groups (overall survival above or below median) were more significant in the CV-Group Lasso and the P-Group Lasso (*p*-value < 0.0001) compared to CV-Lasso and P-Lasso (Fig. 2d).

### Simulation study results

**Fig. 3:**
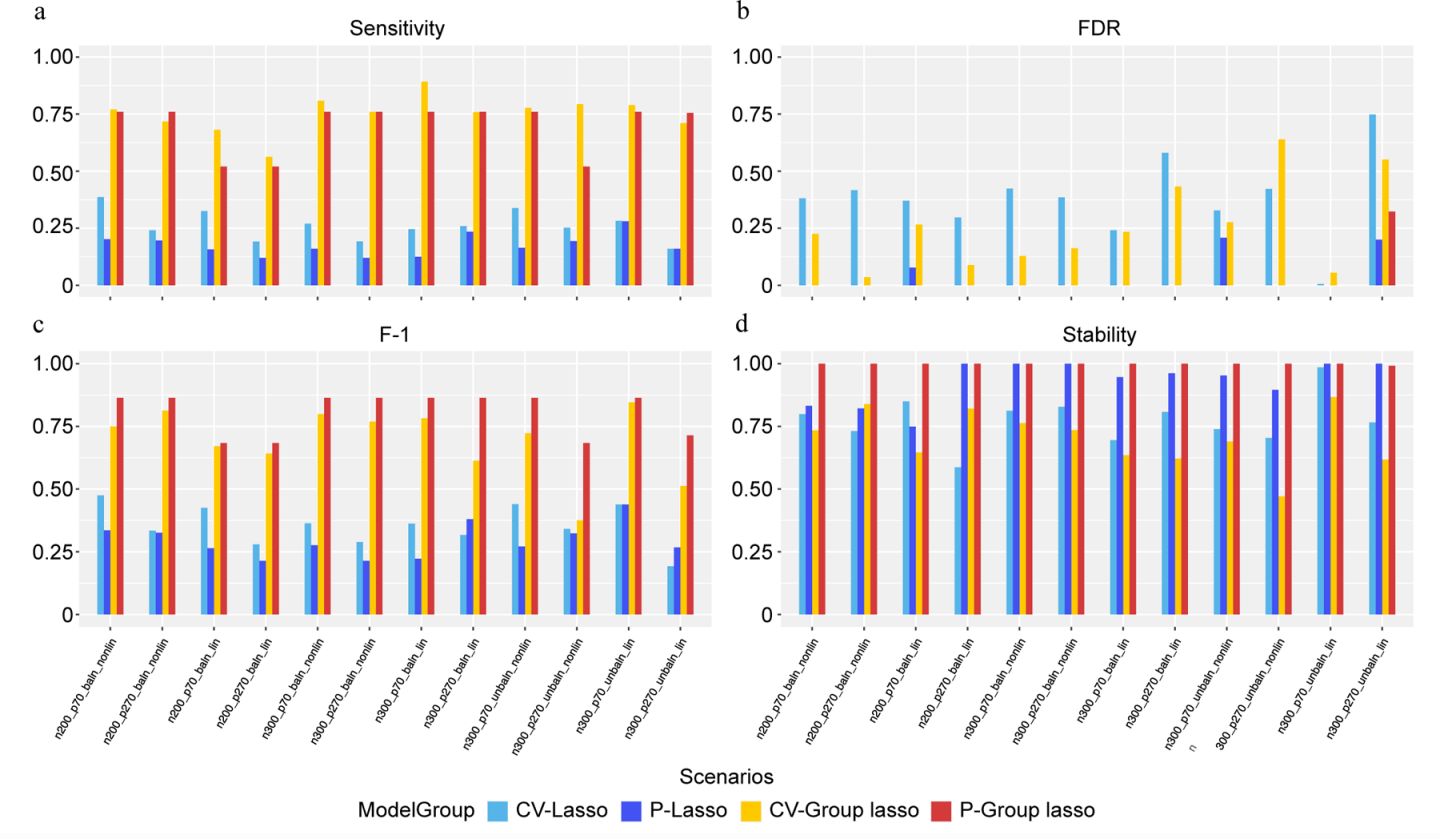
Performance evaluation based on simulation. (a) Sensitivity. (b) FDR. (c) F-1. (d) Stability. In each panel, x-axis stands for different simulation scenario listed in Table 3 and color-coded for different approaches.

An extensive simulation study was conducted to assess the performance of the four models, using the parameters outlined in Supplementary Table S1. The simulation scenarios are detailed in Table 3, with additional parameters provided in Supplementary Fig. S2. The observations indicate that Group Lasso models consistently exhibit higher sensitivity in identifying causal variables compared to Lasso models, with CV slightly outperforming permutation-assisted parameter tuning regardless of the Lasso or Group Lasso approach (Fig. 3a). Notably, permutation-assisted parameter tuning demonstrates superior FDR results for both Lasso and Group Lasso models (Fig. 3b). Specifically, the P-Group Lasso model shows improved performance across F1 scores (Fig. 3c) and stability (Fig. 3d) in all scenarios compared to CV-Group Lasso, with a notably lower FDR. Conversely, P-Lasso and CV-Lasso models exhibit poorer performance across all metrics compared to P-Group Lasso and CV-Group Lasso, except for FDR, where results vary by scenario. Interestingly, P-Lasso and P-Group Lasso models demonstrate the ability to extract causal features without any false positives in certain scenarios, aligning with the lower false positive rates associated with permutation-assisted tuning. Furthermore, increased model stability was observed with P-Lasso and P-Group Lasso, a critical feature in biomedical settings. While CV-Lasso and P-Lasso models extract top network property features regardless of underlying grouping structures, CV-Group Lasso and P-Group Lasso models consistently identify top network properties across all grouped variables. Overall, the models exhibit robustness across various simulation scenarios, including sample size, number of features, balance of outcome interest, and linear vs nonlinear relationships among causal variables.

## Conclusions and discussion

This paper introduces a novel approach to prioritize the heterogenous TCR network properties that are associated with a binary response of interest to identify TCR network properties as the prognostic features or predictive markers in high-throughput TCR sequencing data of clinical samples. The heterogeneous network properties are first aggregated to the homogeneous network features. The present method utilizes a group Lasso model, integrating a group structure to facilitate efficient model fitting and generalization to various response types such as time-to-event, multi-class categorial, and quantitative responses. Additionally, pseudo-values are introduced as known negatives to further enhance selection performance by reducing the false discovery rate and increase the stability of selection. When comparing the proposed P-Group Lasso model result to the two-sample comparison results (Supplementary Table S2), some consistency with the identified properties are observed (e.g., Diameter Length, Eigen Centrality, Central Eigen) using the proposed method. Moreover, the proposed method selected less properties than t-test (4 vs 6) which might indicate its advantages in reducing the false positives, as was observed in simulation studies. This approach has the potential to develop markers from network topological structures to predict the responses.

While the proposed method was specifically applied to TCR network analysis, its versatility extends to a wide range of genetic and medical research data, including genomic, transcriptomic, epigenomic, and proteomic data, with or without a natural group structure. If the features come with a natural group structure (e.g. pathway, multiple class categorical features), the present method can help to prioritize the group associated with the response. If the features don’t have a natural group structure, one can also be defined by letting highly correlated variables form a group. By prioritizing relevant groups associated with the response, the present method enhances interpretability, computational efficiency, and reliability of downstream analyses. It can filter out irrelevant noise variables, prevent overfitting, and facilitate the discovery of meaningful biological insights. Moreover, the present approach can be generalized to various outcome types, including continuous and time-to-event outcomes, beyond the binary setting assumed in this paper.

However, there are two major limitations. Firstly, within each repertoire for each patient, there are numerous clones and hundreds of clusters, each with its node or cluster-level properties. Therefore, there are thousands of values per property per patient. Currently, this complexity is addressed by using summary statistics (such as mean, median, or maximum), which may not adequately represent the data variation. Other distribution features (e.g. percentiles) could also be derived and fed into the proposed method similarly. Secondly, Lasso or Group Lasso are both based on linear models. Though regularization and permutation-assisted tuning were introduced, the performance might be compromised when the true relationship deviates from linear. Future work could involve feature engineering on network properties and extending the linear regression model to a nonlinear one to overcome this limitation.

## Supplementary Material

Supp Table 2

Supp Fig 1

Supp Table 1

Supp Fig 2

## Figures and Tables

**Table 1.** Parameters used for feature selection methods.

Network properties	Description	Function
Count at baseline	Summation of the TCR counts of a given membership at baseline.	sum(input_data[input_datamembership == membership_id, Count_baseline])
Count post treatment	Summation of the TCR counts of a given membership post treatment.	sum(input_data [input_data$membership == membership_id, Count_post_treatment])
Cluster size	The number of node in a given membership.	table(input_data $membership)
Diameter length	The length of the longest geodesic.	get_diameter(net, directed = F)
Assortativity	The assortativity coefficient which is positive if similar vertices (based on some external property) tend to connect to each, or negative otherwise.	assortativity_degree(net, directed = F)
Transitivity	The probability that the adjacent vertices of a vertex are connected.	transitivity(net, type = "global")
Density	The ratio of the number of edges and the number of possible edges.	edge_density(net, loops = F)
Degree centrality	Graph centralization based on the degrees of vertices.	centr_degree(net, mode = "in", normalized = T) $centralization
Closeness centrality	Graph centralization based on the closeness of vertices.	centr_clo(net, mode = "all", normalized = T) $centralization
Eigenvector centrality	Graph centralization based on the eigenvector centralities of positions v within it.	eigen_centrality(net, directed = F, weights = NA) $value
Central Eigenvector	Graph centralization based on the Eigenvector centrality of vertices.	centr_eigen(net, directed = F, normalized = T) $centralization

**Table 2.** TCR network properties and derived network property features.

Network properties	Network property features
Node count	Min, Q1, Median, Mean, Q3, Max
*Count pre infusion	**Min, Q1, Median, Mean, Q3, Max**
Count dose 2	Min, Q1, Median, Mean, Q3, Max
*Diameter length	**Min, Q1, Median, Mean, Q3, Max**
Assortativity	prob(NA), Min, Q1, Median, Mean, Q3, Max
Transitivity	prob(NA), Min, Q1, Median, Mean, Q3, Max
Density	Min, Q1, Median, Mean, Q3, Max
Degree centrality	Min, Q1, Median, Mean, Q3, Max
Closeness centrality	prob(NA), Min, Q1, Median, Mean, Q3, Max
*Eigenvector centrality	**Min, Q1, Median, Mean, Q3, Max**
*Central Eigen	**prob(NA), Min, Q1, Median, Mean, Q3, Max**

* Properties/property features in bold font are considered as the causal properties/features in simulation studies.

**Table 3.** Simulation scenarios.

Scenario #	*n	**P			***Balanced data	****Linear/non-linear
		Causal	Non-causal	Additional		
n200_p270_baln_lin	200	25	45	200	Balanced	Linear
n200_p270_baln_nonlin	200	25	45	200	Balanced	Non-linear
n200_p70_baln_lin	200	25	45	NA	Balanced	Linear
n200_p70_baln_nonlin	200	25	45	NA	Balanced	Non-linear
n300_p270_baln_lin	300	25	45	200	Balanced	Linear
n300_p270_baln_nonlin	300	25	45	200	Balanced	Non-linear
n300_p270_unbaln_lin	300	25	45	200	Imbalanced	Linear
n300_p270_unbaln_nonlin	300	25	45	200	Imbalanced	Non-linear
n300_p70_baln_lin	300	25	45	NA	Balanced	Linear
n300_p70_baln_nonlin	300	25	45	NA	Balanced	Non-linear
n300_p70_unbaln_lin	300	25	45	NA	Imbalanced	Linear
n300_p70_unbaln_nonlin	300	25	45	NA	Imbalanced	Non-linear

* n: # of simulated patients.

** P: # of simulated signals (causal TCR network property features, non-causal TCR n/w property features, additional correlated multivariate noise signals).

*** Balanced data: denotes the simulated dataset has balanced proportion of shorter and longer survival groups.

**** Linear: denotes the simulated response variable Y consist of a linear combination of the causal variables. Non-linear: denotes the simulated variable Y consists of linear and interaction terms generated using the causal variables.

**Table 4.** Results from real data analysis. The table lists the network properties and corresponding network property features selected by each approach.

Network properties	CV-Lasso*	P-Lasso	CV-Group Lasso**	P-Group Lasso**
Count pre infusion	Max	Max	All	All
Count dose2	–	–	All	All
Node count	–	–	All	–
Diameter length	Max	Max	–	–
Assortativity	–	–	–	–
Transitivity	–	–	–	–
Density	–	–	–	–
Degree centrality	–	–	–	–
Closeness centrality	–	–	–	–
Eigenvector centrality	Max	Max	All	All
Central Eigen	Max	–	–	–

* The network property features extracted using CV-Lasso model are used as the causal variables for simulation study.

** The value 'All' represents the entire set of descriptive summary statistics derived from the TCR network property.

## Data Availability

R codes are available on GitHub (https://github.com/ShilpikaB/Prioritizing-Network-Properties-of-T-Cell-Receptors/blob/main/README.md).
