# Supplementary material for: TCR-NP: a novel approach to prioritize T-cell Receptor repertoire network properties
Source: Stat Innov. Author manuscript; Available in PMC 2025 Mar 6. (PMC11884733)
Supplement: Supp Fig 1 [file NIHMS2058198-supplement-Supp_Fig_1.pdf]

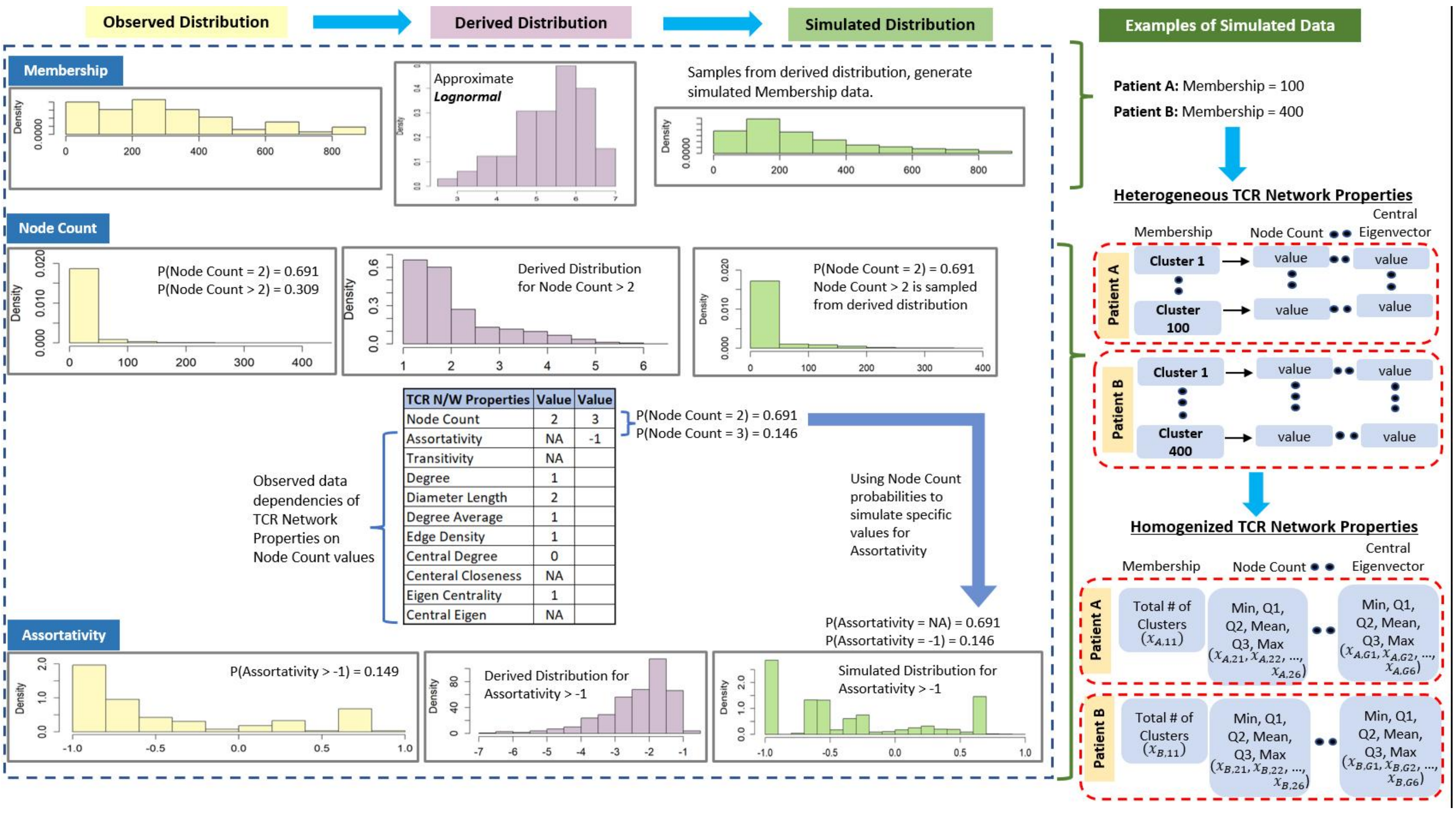

TCR N/W Properties	Value	Value
Node Count	2	3
Assortativity	NA	-1
Transitivity	NA	
Degree	1	
Diameter Length	2	
Degree Average	1	
Edge Density	1	
Central Degree	0	
Centeral Closeness	NA	
Eigen Centrality	1	
Central Eigen	NA	

Supplementary Figure 1: Simulation strategy step 1: Network property feature simulation.
