# Supplementary material for: TCR-NP: a novel approach to prioritize T-cell Receptor repertoire network properties
Source: Stat Innov. Author manuscript; Available in PMC 2025 Mar 6. (PMC11884733)
Supplement: Supp Table 1 [file NIHMS2058198-supplement-Supp_Table_1.pdf]

Supplementary Table 1 Parameters for Data Analysis

	Model	Hyperparameter Tuning Method	Parameters	Comments
**Real Data Analysis**	Lasso[*]	Cross-Validation	k-Folds=5	Used minimum lambda
		Permutation-assisted tuning	# of Permutation Copies (K)=50<br>Threshold (τ)=0.25 (25%)	
	Group[**] Lasso	Cross-Validation	k-Folds = 5	Used minimum lambda
		Permutation-assisted tuning	# of Permutation Copies (K)=50<br>Threshold (τ)=0.25 (25%)	

	Model	Hyperparameter Tuning Method	Parameters	Comments
**Simulated Data Analysis**	Lasso[*]	Cross-Validation	k-Folds=5	Used minimum lambda
		Permutation-assisted tuning	# of Iterations=100<br># of Permutation Copies (K)=50<br>Threshold (τ)=0.5 (50%)	
	Group[**] Lasso	Cross-Validation	k-Folds=5	Used minimum lambda
		Permutation-assisted tuning	# of Iterations=100<br># of Permutation Copies (K)=50<br>Threshold (τ)=0.5 (50%)	

*Lasso analysis was performed using *lasso* R package (version 4.1.3)

** Lasso analysis was performed using *glasso* R package (version 1.5.1)
