# Supplementary material for: TCR-NP: a novel approach to prioritize T-cell Receptor repertoire network properties
Source: Stat Innov. Author manuscript; Available in PMC 2025 Mar 6. (PMC11884733)
Supplement: Supp Fig 2 [file NIHMS2058198-supplement-Supp_Fig_2.pdf]

Simulating Response Variable

[illegible]

[illegible] Z_1, Z_2, Z_3, Z_4 [illegible]

[illegible]	[illegible]	[illegible] 1 [illegible] 1*)	[illegible] 2 [illegible] 2*)	[illegible] 3 [illegible] 3*)	[illegible] 4 [illegible] 4*)
Z_1	Count Pre Infusion	$q_{45}(0.8)$	$q_{55}(0.45)$	$q_{65}(0.75)$	$q_{80}(0.6)$
Z_2	Diameter Length	$q_{60}(0.8)$	$q_{80}(0.95)$	$q_{85}(0.9)$	$q_{95}(0.87)$
Z_3	Eigenvector Centrality	$q_{75}(0.9)$	$q_{80}(0.8)$	$q_{90}(0.9)$	$q_{95}(0.7)$
Z_4	Central Eigen	$q_{55}(0.65)$	$q_{70}(0.7)$	$q_{85}(0.5)$	$q_{90}(0.45)$

*Weights are rescaled to sum to 1

[illegible]

[illegible]:

$$\eta_\alpha(\boldsymbol{Z}) = 0.8Z_1 + 0.99Z_2 + 0.9Z_3 + 0.85Z_4$$

[illegible]:

$$\eta_\alpha(\boldsymbol{Z}) = 0.8Z_1 + 0.99Z_2 + 0.9Z_3 + 0.85Z_4 + 0.015Z_1Z_2 - 0.025Z_2Z_3 + 0.03Z_3Z_4$$

[illegible]

$$\Pr(y = 1) = \frac{\exp\{\eta_\alpha(\boldsymbol{Z})\}}{1 + \exp\{\eta_\alpha(\boldsymbol{Z})\}}$$

Supplementary Figure 2: Simulation strategy step 2: Outcome simulation.
